# A Pocket-Textured Surface for Improving the Tribological Properties of Point Contact under Starved Lubrication

**DOI:** 10.3390/ma14071789

**Published:** 2021-04-05

**Authors:** Wei Wang, Wenhan Zhao, Yang Liu, Hui Zhang, Meng Hua, Guangneng Dong, Hon-Yuen Tam, Kwai-Sang Chin

**Affiliations:** 1 School of Mechanical Engineering, Institute of Design Science and Basic Components, Xi’an Jiaotong University, Xi’an 710049, China; wangweixijiao@stu.xjtu.edu.cn (W.W.); xjtuzwh@163.com (W.Z.); lyly000@stu.xjtu.edu.cn (Y.L.); 2 Department of Mechanical Engineering, City University of Hong Kong, Hong Kong 999077, China; jiaomingHua@gmail.com (M.H.); hon.y.tam@cityu.edu.hk (H.-Y.T.); mekschin@cityu.edu.hk (K.-S.C.)

**Keywords:** starved lubrication, pocket-textured surface, tribology

## Abstract

This paper reports a novel pocket-textured surface for improving the tribological properties of point contact under starved lubrication by possibly storing and releasing oil, and homogenizing the surface contact pressure. The ball-on-disk experimental results confirmed the coefficient of friction (COF) and wear reduction effect of such pocket-texturing. The maximum reduction rate was 40% compared with a flat surface under the same operating conditions. Analyses on experimental results attributed the oil storage effect and enhanced the secondary lubrication effect within the starved lubrication state, to become the main mechanism. In addition, the plate elasticity and the Hertzian contact principles were employed to estimate the pressure and the load acting on the surface. The experimental results and numerical analysis substantiated the design of pocket-textured surface, making it likely to enlarge about 50% of contact surface and to reduce 90% of equivalent stress in comparison to those of conventional surfaces.

## 1. Introduction

Mechanical components, such as gears, cams and rolling-element bearings, commonly have non-conformal contacts, which usually result in, according to the Hertzian contact theory and mixed elastohydrodynamic lubrication, high-contact pressure on the connecting locations [1] and oil-deprivation between interfaces [2]. Such a phenomenon is generally connected with a sharp reduction in the film thickness to just a few nanometers, which raises the severity of wear and friction. This should be properly alleviated to maintain the durability of the operating/sliding friction pairs [3,4,5] problem associated with how lubrication oil behaves and is consumed in practice [4]. Starvation is originally determined by the lubricant failing to replenish the relative motion track [5]. Some researchers defined the degree of starvation as a function of the distance between the inlet meniscus and the Hertzian contact [6,7,8,9,10]. Some researchers showed that the cause of starvation in point contact systems was a result of inadequate replenishment of lubricant fluid [11,12,13,14,15].

Surface texturing has been found to be an important technique for tribological improvement under the state of starved lubrication since the 1960s [16], and the primary mechanism is secondary lubrication [17]. A study on surface texturing by Sorin-Cristian et al. [18] suggested that oil could be transferred from an inlet to an outlet of contact to alleviate both lubricant starvation and friction increment when a solid counterpart passes onto pockets. By utilizing channels to replenish the oil in point contacts, Fadi Ali [5] found a significant improvement in reducing friction. The previous literature has focused specifically on reducing friction and wear between two interfacial surfaces, which fundamentally involves the design of “self-adaptive surfaces” that have the feasibility to change their geometry for mutual adaptation to the surface profile between the mating surfaces, according to their supported load-carrying [19]. For example, micro-dents on the surface, with proper dimensions and edges, reduce the interaction between asperities by emitting some amount of lubricant in the contact, which results in enhancing the film thickness, contact fatigue life and wear resistance, and reduces the friction. On the other hand, a reduction in film thickness with deep micro-dents was investigated under a sliding motion due to the induction of cavitation and the increase in pressure fluctuation in the vicinity of micro-dents [20,21,22,23,24]. However, there is a lack of studies investigating the use of textured surfaces for a better lubricating performance, and testing to enhance the texture oil storage function. Moreover, for the previous texture design, the textures are independent and the depth is limited; therefore, the effect of the oil storage, and the release of lubricant and secondary lubrication, is not obvious. In this paper, a pocket-textured surface is proposed to improve the friction performance in starved lubrication, which is based on elastic deformation and hydrodynamic flow for suitably diminishing friction and wear at non-conformal reciprocating contact. Such pocket-textured structures store lubricant and release it during sliding, thus refilling lubricant to the contact area in time to avoid oil starvation.

In addition, the hardness of the softer surface is an essential factor to determine the wear for contact interfaces. When the hardness/elastic modulus of a friction interface is constant, the other friction interface with a relatively high hardness/elastic modulus may lead to stress concentration, which may imply severe wear. However, the abrasive wear of the softer surface will increase significantly [25]. Hence, the ideal frictional material may have a hard surface and a soft substrate, which is one of the targets of metal heat treatment [26,27] and some coating technologies [28,29]. In our proposed pocket-textured surface, these pockets in resin material are aimed at uniforming the contact stress to reduce severe wear. At the same time, the steel up-layer maintains a hard surface to reduce abrasive wear.

By combining the secondary lubrication effect and homogenizing the contact stress without reducing the surface hardness, the pocket-textured surface is designed to improve the tribological performance. This research has a bright prospect for a future surface-textured design with a 3D porous structure.

## 2. Materials and Methods

### 2.1. Materials Preparation

**Figure 1:**
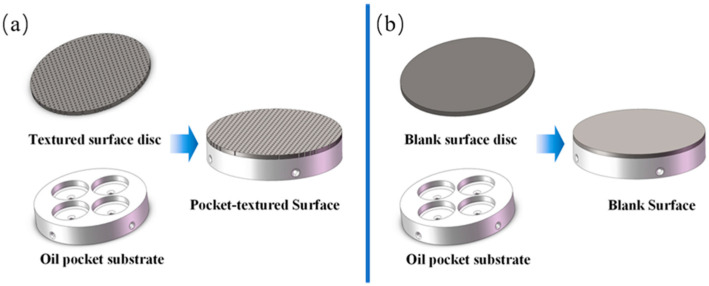
The configuration diagram of the (**a**) pocket-textured surface and (**b**) blank surface.

As illustrated in Figure 1, the configuration of the pocket-textured surface consists of: (i) a circular disc with holes drilled through; (ii) an acrylonitrile butadiene styrene component with four oil pockets and horizontal channels for supplying oil to individual pockets. In order to make a contrast and maintain the comparability of the samples, the comparison sample is composed of a disc without holes and an oil pocket substrate.

**Figure 2:**
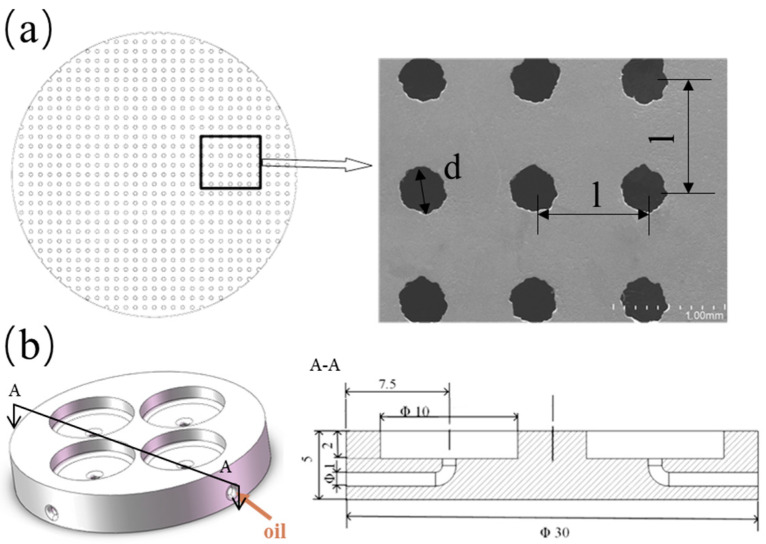
(**a**) Schematic diagram of the textured surface and the SEM of the textured surface; (**b**) the model of the pocket substrate (the unit labeled is in mm).

The disc surface is uniformly textured with through-holes (Figure 2a) fabricated by an electric spark machine. The material of the disc is stainless steel (HV 210). These through-holes have a diameter of 400 μm and the area ratio is 12.5%.

The pocket substrate shown in Figure 2b is made of Acrylonitrile Butadiene Styrene. The sample, which has a diameter of 30 mm and a thickness of 5 mm, was printed by a Lite 450, SLA light-curing 3D-printing apparatus (Union Tech., Dongguan, China). The individual oil pockets were designed with a diameter of 10 mm and a depth of 2 mm. Four channels were uniformly distributed with a diameter of 1 mm and a length of 10 mm below the pockets, which deliver oil to lubricate the pockets.

The textured surface and the oil pocket substrate were glued together by cyanoacrylate after both being ultrasonically cleaned and blow-dried. A piece of 5 mm-thick flat plate was then laid on the top of the glued assembly and a 1 kg weight was placed on it. Each glued assembly was dried for at least 12 h before the experimental tests. The pocket-textured surface and the blank surface were then polished carefully and repetitively by 800# and 1200# grain-sized sandpapers, and by a single-sided raised flannelette, to keep the surface roughness (Ra) within 0.15 μm throughout. They were cleaned in ethyl alcohol with an ultrasonic wave concussion for 10 min to remove any residual pollutant. Finally, they were blow-dried. The pocket-textured surface showed in Figure 2a was observed under a scanning electron microscope (Japanese-made SU-8020, Hitachi, Tokyo, Japan). It can be seen that the circular texture shape has a certain deviation from the circular shape, and there is no burr on the textured edge after polishing.

### 2.2. Friction Test

**Figure 3:**
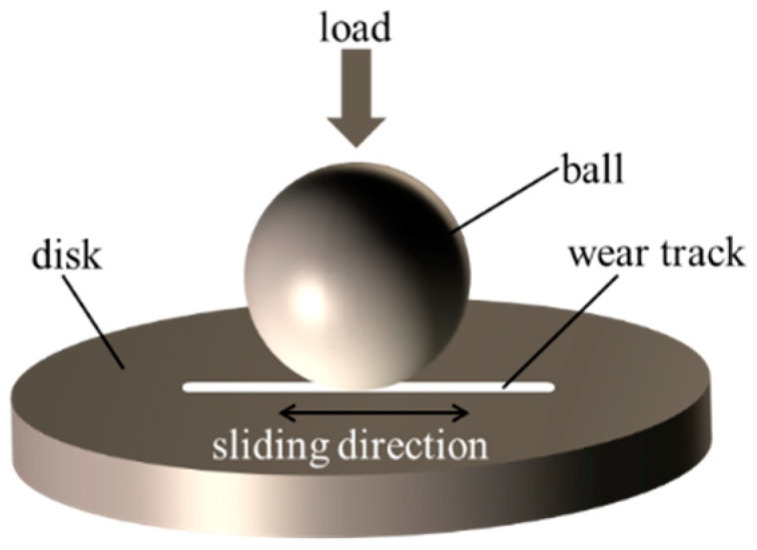
Schematic of the reciprocating test.

Friction performance of the pocket-textured surface was investigated using a ball-on-disc tribo-meter (UMT-2, CETR Corporation Ltd., Campbell, NC, USA) showed in Figure 3. The GCr15 ball has a diameter of 9.5 mm, and a hardness of HRC 62. Tests were performed under reciprocating speeds in a range of 0.5–2.0 Hz and applied loads of 2–15 N. Before starting the tribological test, oil was supplied to the pockets through the connecting pipes fastened to the oil channels until the pockets were full. The oil-pocket-textured surface sample was fully pre-filled before the initiation of any test. The oil was drained to wet the surfaces for performing the starved lubrication. Their tribological behaviors were compared with the blank surface. The lubricant used in all experiments was white oil, which has a density 806 kg/m^3^ and a kinematic viscosity 32 mm^2^/s at 40 °C. The reciprocal sliding length was set at 6 mm. Each test was conducted with 20 min sliding time and generally repeated three times.

The morphology of individually slid surfaces was observed by a scanning electron microscope (SEM), and the wear track profile was detected by a confocal laser scanning microscope (LEXT OLS4000, OLYMPUS, Tokyo, Japan.

### 2.3. Ansys Simulation Preparation

**Figure 4:**
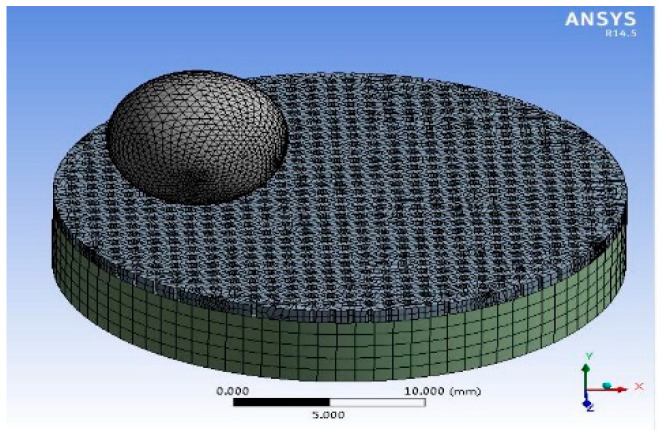
Analysis simulation model of the pocket-textured disc and bearing steel ball.

As the holes of the pocket-textured surface are through-holds, it is difficult to calculate the contact stress of the pocket-textured surface. ANSYS^TM^ software (ANSYS. Inc., South Pittsburgh headquarters, PA, USA) was used to explore the friction mechanism due to the influence of surface deformation/deflection of the pocket-textured surface. The model was established as a proper coordinate system with adequately generated meshes (Figure 4). Furthermore, the model was coded for the forces in the experiments, respectively. Its solution gave corresponding values of total deformation and equivalent stress. To better study the lubrication principle, the Hertzian contact is used for the calculation of the blank surface.

## 3. Results

### 3.1. Tribological Properties of under Different Sliding Speeds

The sliding speed of the friction pair determines the momentum of the lubricant in the oil-pocket and also determines the frequency of contact between the ball and the textured surface. Its influence on the tribological properties of the pocket-textured surface and the blank surface against the steel ball under the load of 2 N, was investigated at reciprocal speeds of 0.5 Hz, 1.0 Hz, 1.5 Hz and 2.0 Hz, respectively.

**Figure 5:**
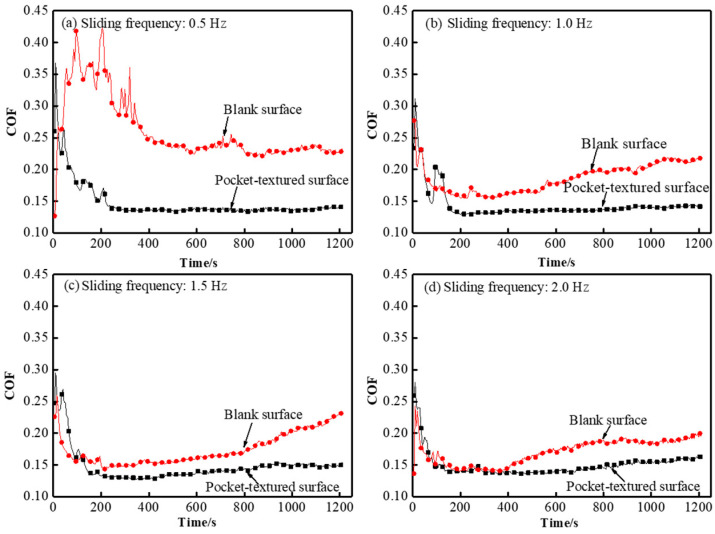
Coefficient of friction (COF) comparison of pocket-textured surface and the blank surface under different sliding frequencies of (**a**) 0.5 Hz, (**b**) 1.0 Hz, (**c**) 1.5 Hz and (**d**) 2.0 Hz with a constant load of 2 N.

Figure 5 illustrates the behavior of the coefficient of friction (COF) under the load of 2 N. The COFs of the pocket-textured surface are lower than those of the blank surface at different speeds under the load of 2 N. The blank surface had a much higher COF in the beginning, and a longer seating time than the pocket-textured surface. Furthermore, the curves of the pocket-textured surface were much smoother than those of the blank surface and the surface shown in the results of previous research [30].

**Figure 6:**
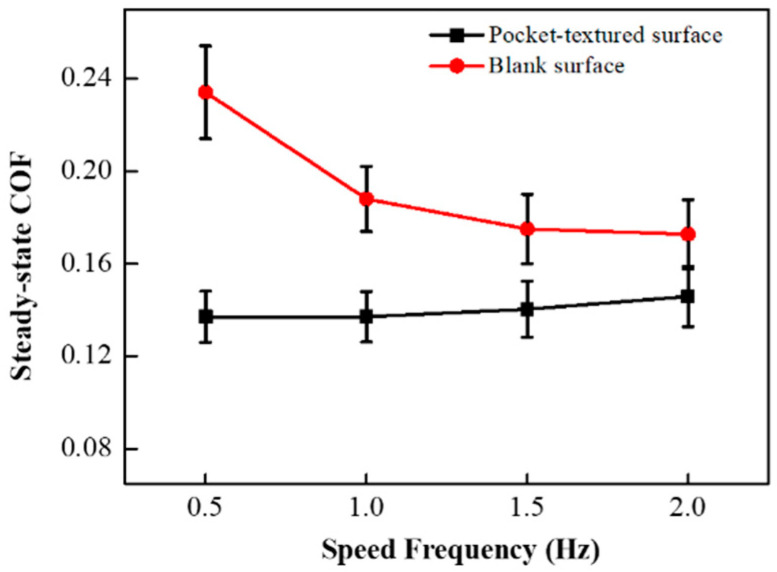
Steady-state COF of the pocket-textured surface and blank surface under different sliding frequencies while the load is kept at 2 N.

The steady-state COFs for the pocket-textured surface are ranging from 0.137 to 0.145 for sliding speed, increasing from 0.5 Hz to 2.0 Hz (Figure 6), which are much lower than the COF obtained by the same materials [31]. This suggests that the pocket-textured surface has more efficient lubrication behaviors than the blank surface. The steady-state COF of the pocket-textured surface rises within a small range, while the steady-state COF of the blank surface decreases greatly with the increase in speed.

**Figure 7:**
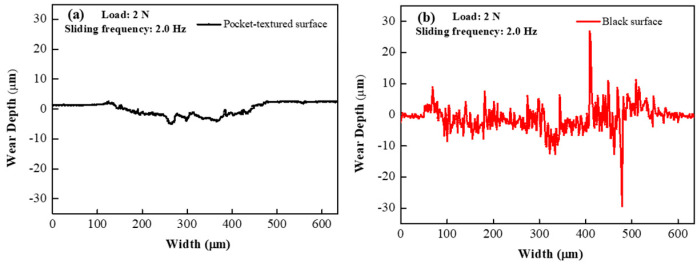
Cross-sectional profiles for (**a**) the pocket-textured surface and (**b**) the blank surface under the load of 2 N and speed frequency of 2 Hz.

The steady-state COF of the pocket-textured surface and the blank surface are close under the load of 2 N and the speed frequency of 2 Hz. The two types of surface topographies of the wear tracks formed are shown in Figure 7. The wear scratch of the pocket-textured surface is observed, with a depth of 5 μm and width of 300 μm, while the deep wear scratch is observed on the blank surface, with a depth of 20 μm and width of 500 μm. The wear track of the blank surface is much deeper and wider than those on the pocket-textured surface. The wear track of the pocket-texture surface is also more shallow than the result that Aaron Greco showed [32].

### 3.2. Tribological Properties under Different Loads

In order to better simulate the actual working conditions, the test adopts a sliding frequency of 0.5 Hz that guarantees boundary lubrication. The tribological properties of the pocket-textured surface and the blank surface sliding against the steel ball under a sliding speed of 0.5 Hz, and loads of 2 N, 5 N, 10 N and 15 N were studied.

**Figure 8:**
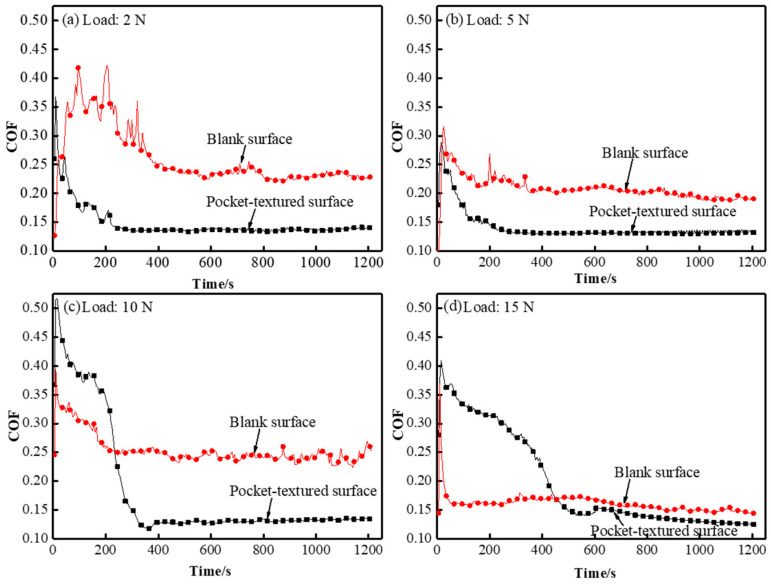
COF comparison of the pocket-textured surface and the blank surface under different loads of (**a**) 2 N, (**b**) 5 N, (**c**) 10 N and (**d**) 15 N with a constant sliding frequency of 0.5 Hz.

A comparison of the transient COF can be seen in the curves shown in Figure 8 for both the pocket-textured surface and the blank surface loaded with various loads. The run-in stage and the steady stage of the pocket-textured surface for the 2 N loading are roughly similar to those for the ball on the blank surface in Figure 8a. The spacing between the curve with the pocket-textured surface and that with a blank surface for the case of mating surface is larger. Furthermore, the shorter fluctuation period of COF, with a widened peak plateau when sliding and almost approaching a steady stage, is a sign of effective lubrication compared to the blank surface. When loading is increased to 5 N, the loaded spot normally experiences more severe defecting on the contractual region (Figure 8b). Compared to Figure 8b,c, an increase in the loading from 5 N to 10 N can be seen, changing the run-in nature completely, and giving higher COF for the pocket-textured surface assemblies than the normally-blank specimen assembly. Furthermore, the steady-state for the pocket-textured surface almost overlaps and slightly surpasses that of the blank surface.

**Figure 9:**
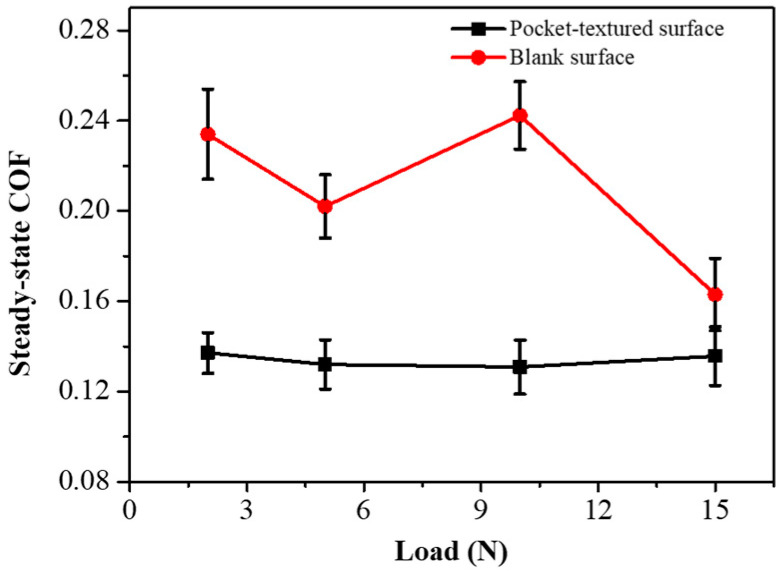
Steady-state coefficients of the pocket-textured surface and blank surface under different loads while the sliding frequency is kept at 0.5 Hz.

Figure 9 presents the steady COF of the two types of friction pairs under different loads. It shows that, under low loads, the pocket-textured surface friction pairs give much lower COF. Generally, the pocket-textured surface friction pairs tend to be advantageous at low-load conditions.

### 3.3. Ansys Simulation

**Figure 10:**
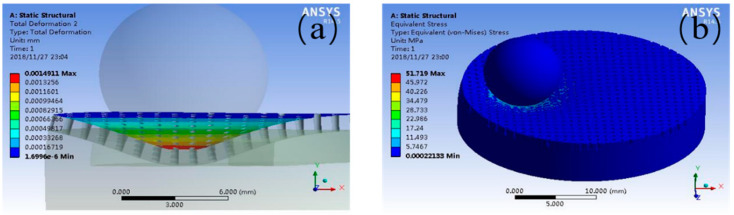
The result of (**a**) the deformation and (**b**) stress of textured surface under the load of 15 N.

The graphical result of the magnified total deformation under the load of 15 N is shown in Figure 10a. In contrast, Table 1 tabulates that the total deformation of the contact on the pocket-textured surface. The maximum press depth often increases with the load application. As it was shown that the deformation of the surface results in squeezing oil from the pockets, such a phenomenon indicates that the pocket-textured surface can provide oil to improve lubrication under starved lubrication conditions. The maximum deformation under different loads is greater than 10 μm, which is theoretically greater than the thickness of the elastohydrodynamic lubrication film. Furthermore, the deformation is also kept within a low magnitude to ensure machine stability.

The result of the equivalent stress under the load of 15 N is shown in Figure 10b. Table 2 tabulates the equivalent stresses of the pocket-textured surface using analysis simulation, and the blank surface with the Hertzian contact simulation, respectively. Equivalent stress is the key factor of the oil lubrication, and it causes stress concentration. The results indicate that the equivalent stresses of the pocket-textured surface have a range in the span of 1% to 5% compared to the blank sample.

## 4. Discussion

### 4.1. Wear Track Analysis

**Figure 11:**
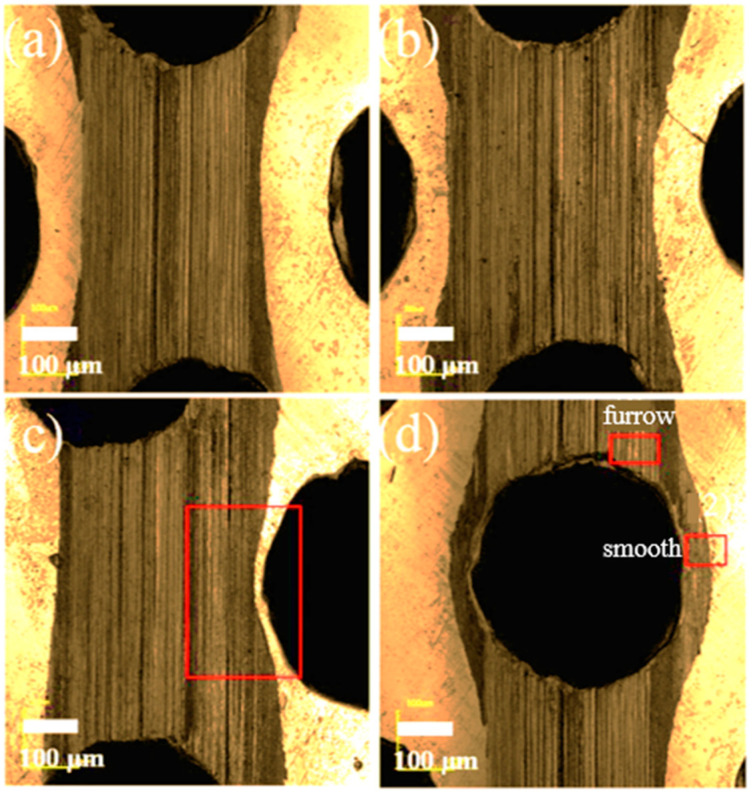
Image of the wear track under (**a**,**c**) the load of 2 N, (**b**,**d**) the load of 5 N, and the sliding speed of 0.5 Hz.

Some typical trends of friction and wear at different friction positions under a sliding speed of 0.5 Hz are illustrated in Figure 8 using a Laser Scanning Confocal Microscopy (LSCM). Almost similar phenomena are observed for imaging in Figure 11a, loaded under 2 N, and in Figure 11b under 5 N, when the ball is sliding through the track between holes. The track that was slid with the lower load is generally much narrower than the track that was slid with a higher load, confirming more severe deformation and wear with an increment of load magnitude. When the ball is running in the middle trace between two neighboring holes (Figure 11c) under the load of 5 N, oil that is squeezed out of the closest hole inclines to flux in cushioning the neighboring region of the hole; it then tends to form a constricted wear track, as in the squared box. The image of the wear track when the ball is running right over the drilled hole under the load of 2 N is illustrated in Figure 11d. The furrow shown in Figure 11c suggests that when oil is squeezed into neighboring troughs, it reduces the wear-off. The barreled-smooth wear track in the vicinity of the hole indicates that the lack of support to the tip of the ball resulted in the weakening region sinking further, hence giving a larger squeezing defection and enlarging the rubbed contact to expand sideways, as labelled. The shallower scratch on the smooth region, compared to the furrowed region, implies that a secondary lubrication by the oil in the troughs is more efficient, and that the lack of support to the ball tip due to the drilling away of materials, resulted in a more severe deformation to the side of the hole transverse section.

**Figure 12:**
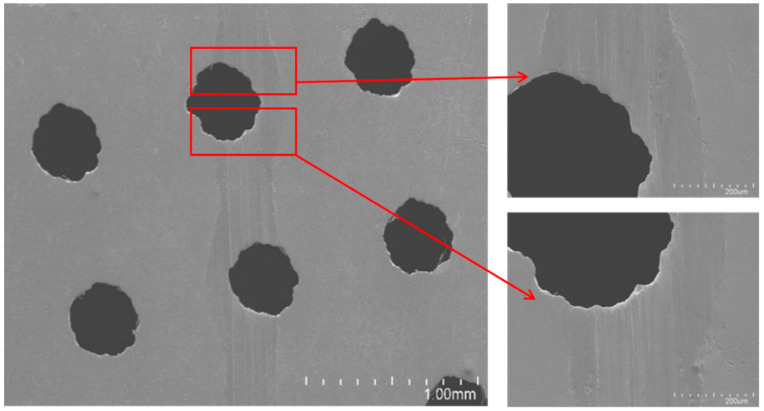
SEM images of wear track under the load of 5 N and the sliding speed of 0.5 Hz.

The wear track under the load of 2 N, and the sliding speed of 0.5 Hz, is further observed by the scanning electron microscope. It seems that it is not the usual strait wear track, but a slightly rubbed path (Figure 12). The scale-like marks along the path suggest the occurrence of a deformation mode of squeezed sliding, and an upstream rim-edge of holes with a smooth downhill slope implies that a certain level of polishing/grinding is taking place. The shining edge with a stepping cliff at its downstream counterpart demonstrates the initial banging of the edge to accumulate materials, which are then cracked off due to the peeling effect when the ball passes the hole-edges. This is attributed to causing the peak and trough values on the corresponding COF curves (Figure 5 and Figure 8).

### 4.2. Discussion

The pocket-textured surface has a lower COF than the blank surface under different sliding speeds. For the pocket-textured surface, the peak and trough section in incipient sliding, where the peak represents its lubricated condition, is almost similar to that of the blank surface, whilst the trough implied that oil lubrication in the interface was established (Figure 5). This diverging phenomenon means the tribological situations are improving when sliding occurs (which often implies more oil in the slide location of the interface). The converging characteristics may suggest that oil is being dragged out from the contacting interface and reducing friction. The COF of the pocket-textured surface only fluctuates in a small range when the speed changes (Figure 6). The further increase in speed tends to swing lubricant from the contact region, thus the lubrication film becomes thinner and more susceptible to cause direct material contact. As a result, COF is increasing slowly with the increase in speed. For the blank surface, the same amount of lubricant is poured on top of the surface. When the speed increases, the COF of the blank surface decreases rapidly. The increase in reciprocal speed generally enhances the momentum of the lubricant and rises with better penetration to the contact region; hence, it results in a slight reduction in COF with an increase in speed. These wear tracks of the pocket-textured surface, and the blank surface under the same load and speed, indicate that the pocket-textured surface has the advantage of reducing wear (Figure 7). This is probably because it can store wear debris in the texture pits [33,34,35].

In Figure 8, the fluctuation for the run-in stage of the pocket-textured surface seems to quickly die down, and there is no obvious peak to be observed, implying that sufficient deformation is taking place on the interfacial surfaces. Reversely, the peak values observed on the curve of the blank-friction pair may be evidence of the accumulation of materials upstreaming on the mating surface to increase shearing force. The larger spacing between the two curves in the run-in stage and its narrow space, implies better tribological behaviors in the first stage of the pocket-textured surface. Their steady stage has very close behaviors as a result of the close, parallel curves in this region. Further increasing loading, interfacial deformation becomes even more severe, which changes the transient characteristics in the run-in stage. Upon increasing the load from 10 N to 15 N, local defection covering the disc is relatively more severe; the initially curving slope around the vicinity leads the outflow of lubricant to the contact area and assists in wetting the incipient stage of sliding. The drilled holes weaken the disc strength and the edge of the holes are likely to initiate more severe peeling. The aforementioned mechanisms resulted in friction behaviors, as plotted in Figure 8d, but showed a slightly higher range of coefficients of friction when compared to the blank surface. High-loading resulted in severe deformation, which diminishes the effect of squeezing out lubricant to wet the rubbing surfaces. The enlargement of contact surfaces increases the possibility of starved lubrication, whilst the diminishing of local pressure provides the following benefits: (i) the surface area contacted is not being closely squeezed, (ii) the asperities in the interfacial contact-surface may not severely deform and lubricant oil can entrap in deforming troughs, etc. Such behaviors indicate that beneficial behaviors for each running condition are different, and can varyd with the loading, sliding speed, drilled holes arrangement, oil pocket locations and thickness of the covering holed disc.

**Figure 13:**
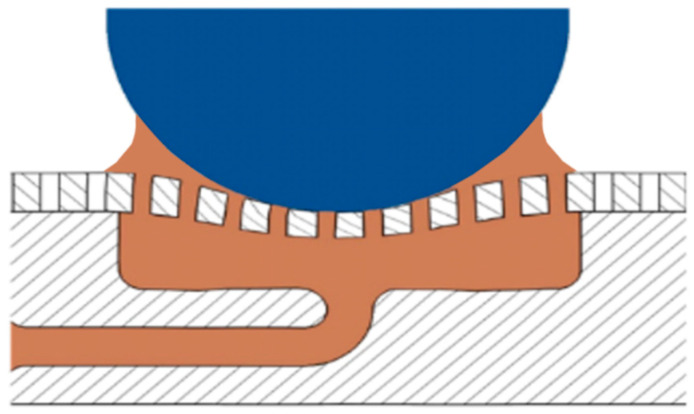
The mechanism of the pocket-textured surface sample under load.

The condition for the pocket-textured surface was slid by the ball shown in Figure 13. As it has hardly any oil in the rubbing surface at the initial state of the run-in, fluctuation of COF on the textured-pocket is mainly due to passing the severely holed and non-holed region alone, with either little or no oil in the regions. Subsequently, filling and squeezing the oil in and out of the troughs in the surface vicinity as the ball moves, plastically deforms the surface. The analysis using elastic mechanics and the Hertzian theory for the ball sliding on the oil-pocket-texture surface suggests that indentation and deflection deformation squeezes the oil out from the pocket. The oil wets the mating surfaces and improves friction performance. When the fluxed-out oil is insufficient to maintain sufficient lubrication, the oil film is then discontinuous and lubrication fails.

The oil-storage cavity of the pocket-textured surface is limited, and it is difficult to add new lubrication during the friction process. Thus, the pocket-textured surface can only improve the short-term oil shortage condition. In the future, we can introduce smart sensors and controllers into this textured design to detect the lack of fuel, provide quantitative fuel, and realize smart fuel supply.

## 5. Conclusions

In this study, the pocket-textured surface is designed, combining oil pockets and texture, which were fabricated by a 3D Printer and an electric sparking machining, respectively. An investigation of the friction performance of the pocket-textured surface under extremely-starved lubrication was conducted. Aiming to understand its involved mechanism, finite element simulation was undertaken to study cases of geometric deformation. The major findings of this research can be categorized into three aspects.

1. The designed pocket-textured surface has better tribological performance under low-load. Different textured surfaces incline to vary the upper-limit of such loads. This is mainly because higher loads tend to severely deform the asperities rapidly in textured surfaces;
2. The designed pocket-textured surface pertains better tribological performance under slow sliding speed, which may provide a means to solve the run-in problem that happened at the start and the instant of the stop;
3. The designed pocket-textured surface, without changing the surface hardness, has great equivalent stress reduction, while it results in smaller deformation compared to the regular surface.

## Figures and Tables

**Table 1:** The results of the deformation of the pocket-textured surface under different loads.

Force (N)	2	5	10	15
Deformation of textured surface (m/10^−8^)	19.9	49.7	99.4	149.1

**Table 2:** The result of the stress of the textured surface and smooth surface.

Force (N)	2	5	10	15
Equivalent stress of textured surface (MPa)	6.89	17.24	34.479	51.719
Stress of smooth surface (MPa)	580	790	1000	1140

## Data Availability

The raw/processed data required to reproduce these findings can-not be shared at this time as the data also forms part of an ongoing study.
